# Screening for *CCNF* Mutations in a Chinese Amyotrophic Lateral Sclerosis Cohort

**DOI:** 10.3389/fnagi.2018.00185

**Published:** 2018-06-29

**Authors:** Danyang Tian, Jiao Li, Lu Tang, Nan Zhang, Dongsheng Fan

**Affiliations:** ^1^Department of Neurology, Peking University Third Hospital, Beijing, China; ^2^Key Laboratory for Neuroscience, Ministry of Education/National Health Commission, Peking University, Beijing, China

**Keywords:** amyotrophic lateral sclerosis, *CCNF* gene, novel mutation, Chinese population

## Abstract

Previous research has identified *CCNF* mutations in familial (FALS) and sporadic amyotrophic lateral sclerosis (SALS), as well as in frontotemporal dementia (FTD). The aim of our study was to measure the frequency of *CCNF* mutations in a Chinese population. In total, 78 FALS patients, 581 SALS patients and 584 controls were included. We found 19 missense mutations, nine synonymous mutations and two intron variants. According to the American College of Medical Genetics and Genomics (ACMG) standards and guidelines for the interpretation of sequence variants, eight variants were judged to be pathogenic or likely pathogenic variants. The frequency of such variants was 2.56% in FALS and 1.03% in SALS. In conclusion, *CCNF* mutations are common in FALS and SALS patients of Chinese origin, and further study is still needed.

## Introduction

Amyotrophic lateral sclerosis (ALS) is a fatal neurodegenerative disease that is characterized as selectively involving the upper and lower motor neurons and partly overlaps with frontotemporal dementia (FTD). Among all cases of ALS, 10% are familial ALS (FALS), while the rest are sporadic ALS (SALS), indicating that genetic factors play an important part in the pathogenesis of ALS (Rowland and Shneider, 2001). Among the known ALS-related gene mutations, *SOD1*, *TARDBP* and *FUS* mutations are common genetic factors that explain a relatively large proportion of FALS and SALS cases, whereas mutations of other related genes, such as *SQSTM1*, *OPTN*, *TUBA4A* and *ARHGEF28*, are less common (Ma et al., 2014; Li et al., 2015a,b; Yang et al., 2015). Expanded GGGGCC repeats in *C9orf72* are reported to be a common genetic factor, occurring in 21.7%–57.9% of Caucasian FALS patients (DeJesus-Hernandez et al., 2011; Renton et al., 2011; Majounie et al., 2012). However, gene screening of *C9orf72* in China showed a much lower rate of expanded repeats (Liu et al., 2013; He et al., 2015). Studies in other east Asian countries showed similar results, leaving a large percentage of cases where the explanatory genes are unknown (Ogaki et al., 2012; Jang et al., 2013). This gap indicates that other common genes may exist in this population.

Impairment of the ubiquitin-proteasome system (UPS) is an important pathological mechanism in ALS. It has been discovered that TDP-43 protein is a major component of ubiquitin-positive neuronal inclusions in ALS patients, and mutations in *TARDBP* and *FUS* are both related to the development of such inclusions (Neumann et al., 2006; Kwiatkowski et al., 2009). Recently, *CCNF* mutations were discovered to be related to ALS (Williams et al., 2016). *CCNF* encodes cyclin F, which is a component of an Skp1-Cul1-F-box protein (SCF) ubiquitin-protein ligase complex and mediates protein ubiquitination and proteasomal degradation. That study discovered a missense mutation (p.S621G) with disease segregation in a large family of British ancestry and then screened *CCNF* variants from FALS, SALS and FTD patients in diverse geographic populations; furthermore, they investigated the role of mutated cyclin F in UPS impairment. The CCNF mutant, Ser621Gly, disrupted the Lya48-specific ubiquitylation, resulting in accumulation of RRM2 and TDP-43, which are ubiquitinated proteins (Lee et al., 2017). Cyclin F interacts with p62, the receptor responsible for transporting ubiquitylated substrates for autophagic degradation (Lee et al., 2018). Zebrafish with Ser621Gly mutant showed disruption of axonal outgrowth, suggested a toxic gain-of function mechanism in ALS pathogenesis (Hogan et al., 2017). We investigated the percentage of CCNF mutations in Chinese FALS and SALS patients to find out whether it is a common genetic factor for ALS in the Chinese population.

## Materials and Methods

### Participants

There were 78 FALS patients, 581 SALS patients and 584 controls included in this study. ALS patients were diagnosed as clinically definite, probable or laboratory-supported probable ALS according to the revised El Escorial criteria (Brooks et al., 2000). Patients with definite or probable FALS were included, which is proband having one or more first- or second-degree relative with ALS (Byrne et al., 2011). The controls were healthy, sex- and age-matched people with no personal or FALS history of neurological disorders. We screened all the exomes in 384 control cases, and later, we added 200 control cases, which we screened only for the exomes including pathogenic or likely pathogenic variants. All of the patients underwent regular follow-up visit every 3 months. ALSFRS was used to judge the severity of disease and ΔFS was used to judge the progress (Kaufmann et al., 2005; Kimura et al., 2006). This study was carried out in accordance with the recommendations of the Peking University Third Hospital ethics committee. The protocol was approved by the Peking University Third Hospital ethics committee. All patients and controls gave written informed consent and they all came from the mainland of China.

### Gene Screening and Variation Analysis

Genomic DNA was extracted from peripheral blood leukocytes using a standard salting-out protocol. Amplification of 17 exons and intron-exon flanking regions of the* CCNF* gene were performed using polymerase chain reaction (PCR) analysis with primers designed by the software Primer Premier 5. The primer sequences are listed in a supplementary file (Supplementary Table S1). The PCR products were sequenced by Tsingke Biotechnology Co., Ltd. (Beijing, China). The sequence variants were validated by sequencing both the sense and antisense strands of the amplicons.

### Bioinformatics

All nonsynonymous variants were screened with the dbSNP137, Exome Aggregation Consortium (ExAC), 1000 Genomes Project and NHLBI Exome Sequencing Project (ESP6500) databases to identify previously reported variants. Novel variants were defined as those that were not present in the aforementioned databases. Pathogenic and likely pathogenic variants were identified using the American College of Medical Genetics and Genomics (ACMG) standards and guidelines for interpretation of sequence variants (Richards et al., 2015). The functional properties of the nonsynonymous variants were predicted in silico using SIFT^1^ and PolyPhen-2 software^2^ to assess toxicity. The evolutionary conservation of the mutation sites was analyzed by aligning the amino acid sequences using the UniProt Web site.

## Results

**Figure 1:**
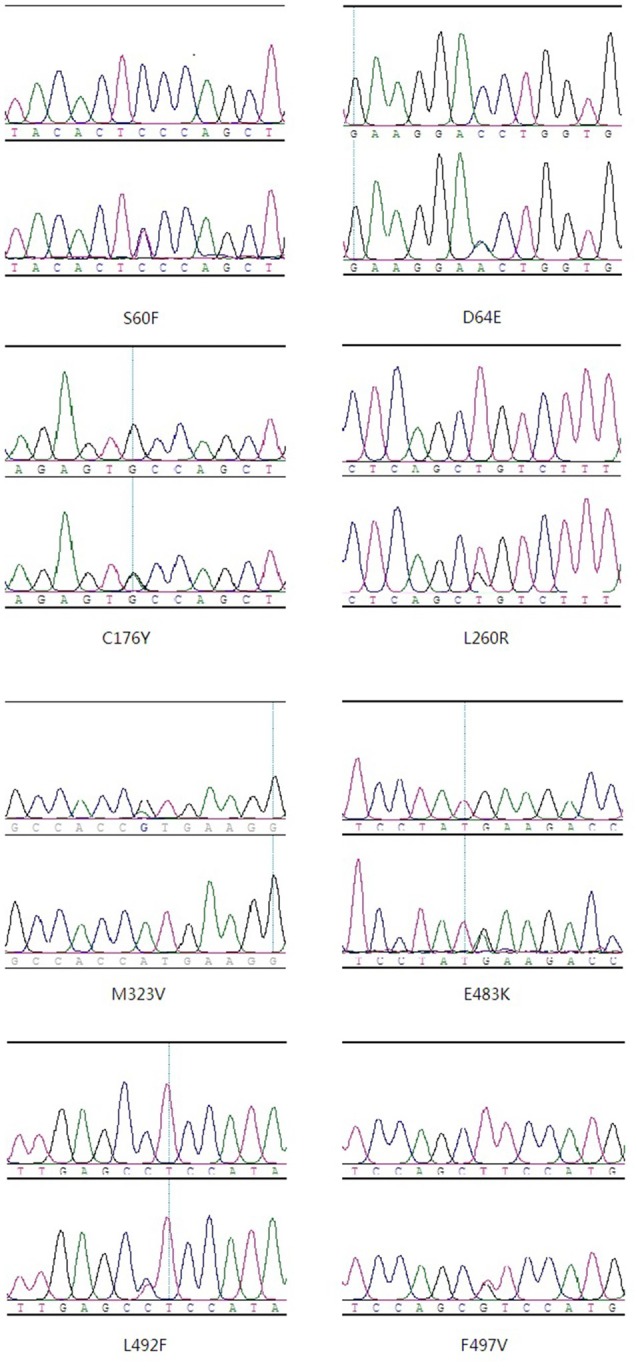
Pathological or likely pathological variants in amyotrophic lateral sclerosis (ALS) patients.

In total, there were 19 protein-altering variants of *CCNF* (all missense mutations, two in FALS, 12 in SALS, two in control, and two in both SALS and controls; Supplementary Table S2) There were seven novel mutations in ALS patients. (Table 1, Figure 1) Two common variants (F604I, R691Q) were present in both patients and controls (common variants were defined as allele frequency >0.01 in our controls or in the 1000 Genomes Project or ExAC databases). There were nine synonymous mutations, including one common variant (c.435C > T). There were two intron variants (insertion or deletion) in ALS patients (Supplementary Tables S3,S4).

**Table 1:** Pathological or likely pathological variants detected in this study.

Exons	Mutations	Nucleotide change	rsID	Case	Control	Public database MAF	SIFT	POLYPHEN
FLAS								
13	E483K	c.1447G > A	rs765151794	1/78	0/584	Absent*	Deleterious	Possibly damaging
14	F497V^#^	c.1489T > G		1/78	0/584	Absent	Deleterious	Probably damaging
SALS								
3	S60F	c.179C > T		1/581	0/584	Absent	Deleterious	Possibly damaging
3	D64E	c.192C > A		1/581	0/584	Absent	Tolerated	benign
5	C176Y	c.527G > A		1/581	0/584	Absent	Deleterious	Possibly damaging
9	L260R^#^	c.779T > G		1/581	0/584	Absent	Deleterious	Possibly damaging
10	M323V	c.967A > G		1/581	0/584	Absent	Deleterious	Possibly damaging
13	L492F	c.1474C > T		1/581	0/584	Absent	Deleterious	Probably damaging

*Was found a singleton in ExAC database. ^#^Was located at the splicing region. Public database includes dbSNP, ExAC, the 1000 genomes project and the GO-ESP.

**Figure 2:**
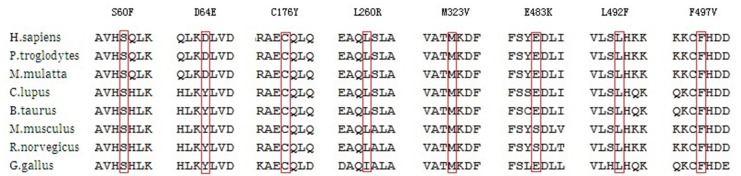
Alignment of the mutations in different species.

As for the two missense variants in the FALS patients, F497V is a novel missense variant in the splicing region and was absent from the public databases, E483K is an existing variant documented as a singleton in the ExAC database. Among the 12 variants in SALS patients, six variants (S60F, D64E, C176Y, L260R, M323V, L492F) were novel, with no entries in the public databases. Here, we discussed the seven novel variants and the E483K variant in FALS patients. Six of the eight variants were located in the functional domain of the *CCNF* gene, while C176Y and L260R were not. Prediction of functional properties by SIFT and PolyPhen-2 showed that seven of the eight variants, excluding D64E, had deleterious or possibly damaging functions. Six of the eight variants were highly conserved among different species, while D64E and E483K were less highly conserved (Figure 2). None of the above variants were found in controls.

For patients with previously discussed eight variants, the average onset age was 52.6 (41–69) years old. The patients who carried those variants included seven males and one female. The mean diagnostic delay was 25 (6–60) months. All of the eight patients presented limb onset. All the patients denied having a history of FTD, neither did they undergo the standardized Edinburgh Cognitive and Behavioral ALS Screen (ECAS). Prognoses of the eight patients varied a lot. Five of the eight patients died, and ΔFS scores were quite different among these patients, ranging from 0.11 to 1.91 (Table 2).

**Table 2:** Clinical data of the pathological or likely pathological variants.

Mutation	Range of onset age*	Site of onset	Diagnostic delay (month)	ALSFRS-R^#^	Dementia	Survival times (month)	ΔFS
S60F	3	Limbs	36	14	No	55	0.94
D64E	6	Limbs	12	40	No	49	0.67
C176Y	6	Limbs	10	42	No	43	0.60
L260R^†^	1	Limbs	6	47	No	52	0.17
M323V	5	Limbs	11	27	No	29	1.91
E483K	4	Limbs	12	45	No	40	0.25
L492F^†^	3	Limbs	53	42	No	92	0.11
F497V^†^	3	Limbs	60	39	No	80	0.15

*Range of onset age:1: 41–45 years old, 2: 46–50 years old, 3: 51–55 years old, 4: 56–60 years old, 5: 61–65 years old, 6: 66–70 years old; ^#^ALSFRS-R was evaluated at the first visit; ^†^Patients were still alive.

**Figure 3:**
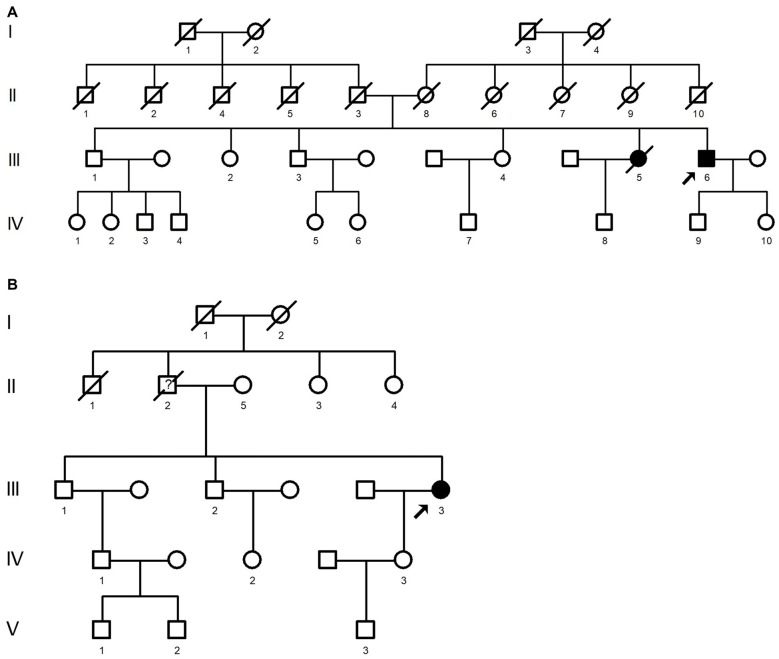
Pedigree carried E483K mutation **(A)** and F497V mutation **(B)**. The arrow on the pedigree represents the proband. The circles denote females, and squares denote males. Affected individuals are noted by black symbols and unaffected individuals are noted by blank symbols. Deceased individuals are noted by a slash symbol.

The E483K mutation was identified in a fALS proband. (Figure 3A: III6). At age 56, he developed weakness and muscle atrophy of left hand. One year later, he developed weakness of lower limbs. His speech was inarticulate. Sucking reflex was positive. The tendon reflex was active. Abdominal reflexes were decreased. His sister (Figure 3A: III5) showed weakness of limbs at age of 35, she was diagnosed as ALS in another hospital and died 5 years after disease onset.

The F497V mutation was detected in another fALS patient. (Figure 3B: III3). She developed slowly progressive right upper limb weakness and muscle atrophy when she was 55 years old. Later she developed dysphagia and choking. The weakness gradually spread to the lower limbs and upper left limb. Five years later, muscle atrophy was appeared in left hand. The tendon reflex was active, except left radial periosteal reflex was absent. Abdominal reflexes were decreased. Babinski’s sign was negative. Electromyogram showed neurogenic damage. Her father (Figure 3B: II2) showed weakness and muscle atrophy of upper limbs at age of 60. At age of 70, he admitted to the hospital because of catching a cold, he tumbled to the ground accidently and passed away.

## Discussion

Recent researches have revealed that *CCNF* mutations are associated with ALS pathogenesis. Our study analyzed CCNF variants in a large Chinese ALS cohort.

According to the ACMG standards and guidelines for the interpretation of sequence variants, we analyzed the *CCNF* mutations that we identified in ALS patients. In total, we found eight missense mutations that met the criteria for pathogenic or likely pathogenic variants. F497V in FALS patients and L260R in SALS patients were identified as pathogenic mutations. Both of them were located in the splicing region, absent from the public databases and the control cases, and predicted as deleterious or possibly damaging variants by SIFT and PolyPhen-2. Additionally, F497V was located in the cyclin C region, which is a functional region. S60F, D64E, C176Y, M323V and L492F in SALS patients and E483K in FALS patients were identified as likely pathogenic variants. S60F and D64E were located in the F-box region, which mediates interaction with other components of SCF ubiquitin-protein ligase complex for ubiquitylation of target substrates. M323V was located in the cyclin N region, E483K and L492F were located in the cyclin C region, and both of the two regions contain a hydrophobic patch that binds the substrate. C176Y was not located in any functional region. but it could also be associated with abnormal protein folding and aggregation. Five of those six, excluding D64E, were predicted to be deleterious or possibly damaging variants by SIFT and PolyPhen-2. All of them were absent from the public MAF databases and were not common in the control patients. Notably, E483K was an existing variant, having been documented as a singleton (MAF = 8.629 × 10^−6^) in the ExAC database. However, the ExAC database contained patients with schizophrenia, a common comorbidity of ALS; therefore, we still thought the evidence moderately supported the pathogenicity of the variant. Since we only obtained the DNA sample in the probands of the FALS, we were unable to judge whether there was a co-segregation in family members. Overall, there were two pathogenic or likely pathogenic variants among 78 FALS patients and six pathogenic or likely pathogenic variants among 584 SALS patients. The explained percentages were 2.56% for FALS and 1.03% for SALS.

We compared the clinical features of patients having pathogenic or likely pathogenic *CCNF* variants with the common feature of ALS patients in this cohort previously published (Chen et al., 2015). For the patients with CCNF mutations, the onset age was later (52.6 vs. 49.8 years old) and men accounted for a higher proportion (male: female 7:1 vs. 1.7:1). There is an obviously higher limb-onset rate (100% vs. 75.1%) compared with the common stage. The diagnostic delay was obviously longer (25 vs. 14 months). The prognosis varied a lot with both rapid progress and slow progress among these patients. In previous research, *CCNF* mutations were found to exist in both ALS patients and FTD patients, which is similar to* TARDBP* and *FUS* mutations. In our study, the relevant patients reported no history or family history of dementia. However, they did not complete a cognitive function assessment; a follow-up study is still needed to test for a connection between our novel *CCNF* variants and cognitive decline.

Previous research has reported a relatively low rate of CCNF mutations in a Chinese ALS cohort, which contains a smaller sample size (269 ALS and FTD patients). In the previous study, an existing heterozygous variant (c.481G4A) was detected in a SALS case with a frequency of 0.6% (Pan et al., 2017). The present study contains a larger sample size (totally 659 ALS patients) and the result is much similar as previously reported in Japan (1.06%), both of which are Asian population (Williams et al., 2016).

In summary, we screened for *CCNF* gene mutations among Chinese ALS patients and found that *CCNF* was exceeded only by *SOD1*, *FUS* and *TARDBP* in the reported frequency of mutations. This finding might, to some extent, fill the gap in our knowledge caused by the low frequency of *C9orf72* in the Chinese population. Further research is still needed in the form of functional studies.

## Author Contributions

DF conceived this study, provided financial support and also responsible for project management. DF and DT designed the study, responsible for preparing and revising the manuscript and had key roles in the study. JL, LT and NZ took part in the design of the study and in sample collection. DF, DT, JL and LT conducted data management. NZ conducted data follow-up. DT and LT undertook data checking. DT, JL and DF undertook statistical analysis.

## Conflict of Interest Statement

The authors declare that the research was conducted in the absence of any commercial or financial relationships that could be construed as a potential conflict of interest.

## Footnotes

^1^http://sift.jcvi.org/

^2^http://genetics.bwh.harvard.edu/pph2/
