# Supplementary material for: Screening for CCNF Mutations in a Chinese Amyotrophic Lateral Sclerosis Cohort
Source: Front Aging Neurosci. 2018 Jun 29;10:185. doi: 10.3389/fnagi.2018.00185 (PMC6034086; doi:10.3389/fnagi.2018.00185)
Supplement: Supplementary file 1 [file Data_Sheet_1.PDF]

TableS1 Primers covering exons, intron-exon boundaries and promoter region.

CCNF-1F	CGGTAAATGAGGCGAGCACA
CCNF-1R	GATCGTTGCCCCAGGAAAGG
CCNF-2F	CATCTTTTGTGGCACAGGGA
CCNF-2R	AGCCAAAGTTTTGTTCTGACAGTT
CCNF-3F	ACCCTACATCTGCCTCTCGT
CCNF-3R	TGTGGGAATGGATCACAGCC
CCNF-4F	TTCTGACTTCTTCAAGTGCTGT
CCNF-4R	CTCCTGAGAGGGAATGGGGA
CCNF-5F	CCATCCTGGAGGGCTCTACT
CCNF-5R	TCCTGACTTTCCAGGGTCCA
CCNF-6F	CATGGGACCCAAAGACAGACA
CCNF-6R	GAGGTTCGATGCCTCCTTGA
CCNF-7F	GTTAGCCAAGATCGTGCCACT
CCNF-7R	CCAAGGCTGGAGGGAGAAAG
CCNF-8F	TGCTGGACAAAGGCGTAGTT
CCNF-8R	CAGTTCCAGTGCTGAACCGA
CCNF-9F	CATTTGGTGCCTGAATGGGC
CCNF-9R	TAAGGGAACTCTATGCCGCC
CCNF-10F	AGCCTTGGTTCCAGGGTAAAC
CCNF-10R	AGCACATGAAGGGCAGGTGAC
CCNF-11F	TCCTAAAGCCTTGGGTCCCT
CCNF-11R	CAGCAGGAAAGCTGAATGCC
CCNF-12F	CATCGGCGTGAACATCATCC
CCNF-12R	TCACTGAAGGTAGGAGCCGT
CCNF-13F	TGAGCTCCAAGAAACTCCACA

CCNF-13R	AATTATAGGCGTGCACCACCA
CCNF-14F	GGGCTGCGTTTATTCTCTTCC
CCNF-14R	TAATGCAGCACACAACTGGC
CCNF-15F	CCCCAAGGACTACAGGCAAG
CCNF-15R	GGAGACAGTGGAGGTTACGC
CCNF-16F	CTGTTCTAGCTTCACCGGCA
CCNF-16R	CCTCTCTCCCCATGTCTGGA
CCNF-17F	CCTTGCCAGAAGCCCAGTTA
CCNF-17R	CTCCATGGTCTATGCAGCGT

Table S2 Missense mutations detected in ALS patients and controls

exons	mutations	nucleotide change	rsID	FALS	SALS	control	public database MAF
3	S60F	c.179C>T		0/78	1/581	0/584	absent
3	D64E	c.192C>A		0/78	1/581	0/584	absent
3	N87K	c.261C>A	rs752507974	0/78	1/581	0/384	0.00007/7 ExAC
5	A146T	c.436G>A	rs749253539	0/78	0/581	1/384	0.000008/1 ExAC
5	C176Y	c.527G>A		0/78	1/581	0/584	absent
9	L260R	c.779T>G		0/78	1/581	0/584	absent
10	M323V	c.967A>G		0/78	1/581	0/584	absent
10	R344K*	c.1031G>A	rs371050277	0/78	1/581	0/584	0.00007/8 ExAC<br>0.00008/1 GO-ESP
12	S432N	c.1295G>A	rs754760067	0/78	1/581	0/384	0.00002/2 ExAC
13	E483K	c.1447G>A	rs765151794	1/78	0/581	0/584	0.000008/1 ExAC
13	L492F	c.1474C>T		0/78	1/581	0/584	absent
14	F497V	c.1489T>G		1/78	0/581	0/584	absent
15	L531R*	c.1592T>G	rs372723774	0/78	1/581	1/384	0.0003/34 ExAC<br>0.0010/5 1000 G
16	F604I*	c.1810T>A	rs118131564	0/78	20/581	14/384	0.0051/579 ExAC<br>0.0044/22 1000 G<br>0.0007/9 GO-ESP
17	G634S	c.1900G>A	rs563777257	0/78	0/581	1/384	0.000010/1 ExAC<br>0.0002/1 1000 G
17	K663R	c.1988A>G	rs771041686	0/78	1/581	0/384	0.000008/1 ExAC
17	R691Q*	c.2072G>A	rs148419125	0/78	4/581	3/384	0.0004/44 ExAC

							0.0004/2 1000 G<br>0.0003/4 GO-ESP
17	R751C	c.2251C>T	rs142532028	0/78	1/581	0/384	0.0003/35 ExAC<br>0.0002/1 1000 G<br>0.0002/2 GO-ESP
17	P756L	c.2267C>T	rs370448527	0/78	1/581	0/384	0.00009/11 ExAC<br>0.0006/3 1000 G<br>0.00008/1 GO-ESP

* were reported in the former research investigated CCNF mutations in ALS patients. Public database includes dbSNP, ExAC, the 1000 genomes project and the GO-ESP.

Table S3 Synonymous mutation detected in this study

exons	nucleotide change	rsID	FALS	SALS	control	public database MAF
5	c.435C>T	rs200978756	0/78	7/581	4/584	0.0001/17 ExAC
						0.0002/1 1000 G
7	c.681A>G		0/78	1/581	0/384	absent
9	c.915C>T		1/78	0/581	0/584	absent
10	c.1089C>T		0/78	0/581	1/584	absent
11	c.1161C>T	rs762782804	0/78	0/581	1/384	0.00002/2 ExAC
12	c.1362C>T	rs373935407	0/78	0/581	1/384	0.00002/2 ExAC
						0.00008/1 GO-ESP
12	c.1377G>A	rs377030852	0/78	1/581	0/384	0.00002/2 ExAC
						0.00008/1 GO-ESP
13	c.1404G>A		0/78	1/581	0/584	absent
16	c.1755C>T	rs199894140	0/78	1/581	0/384	0.000009/1 ExAC
						0.0002/1 1000 G
17	c.1899C>T	rs200209194	0/78	1/581	0/384	0.00003/3 ExAC
17	c.1944T>C		0/78	1/581	0/384	absent

Public database includes dbSNP, ExAC, the 1000 genomes project and the GO-ESP.

Table S4 Intron variants detected in this study

exons	variants	case	control
14	g.23587_23588insA	1/581	0/584
17	g.27035_27035delG	6/581	0/384
